# Utilization of somatic specialist services among psychiatric immigrant patients: the Norwegian patient registry study

**DOI:** 10.1186/s12913-018-3672-y

**Published:** 2018-11-13

**Authors:** Dawit Shawel Abebe, Jon Ivar Elstad, Lars Lien

**Affiliations:** 1 Department of Nursing and Health Promotion, Oslo Metropolitan University, Oslo, Norway; 2 Norwegian National Advisory Unit on Concurrent Substance Abuse and Mental Health Disorders, Innlandet Hospital Trust, Brumunddal, Norway; 3 NOVA, Oslo Metropolitan University, Oslo, Norway; 4 Department of Public Health, Innlandet University College, Brumunddal, Norway

**Keywords:** Health care, Specialist services, Immigrant, Mental disorder, Circulatory diseases, Infectious diseases, Register study

## Abstract

**Background:**

Amongst psychiatric patients, the leading causes of reduced quality of life and premature death are chronic viral infections and cardiovascular diseases. In spite of this, there are extremely high levels of disparity in somatic healthcare amongst such populations. Little research has explored patterns of healthcare utilisation and, therefore, this study aims to examine the use of somatic specialist healthcare for infectious diseases and diseases of circulatory system among psychiatric patients from different immigrant groups and ethnic Norwegians.

**Methods:**

Register data from the Norwegian Patient Registry and Statistics Norway were used. The sample (ages 0–90+) consisted of 276,890 native-born Norwegians and 52,473 immigrants from five world regions – Western countries, East Europe, Africa, Asia, and Latin America, all of whom had contacts with specialist mental healthcare during the period 2008–2011. Statistical analyses were applied using logistic regression models.

**Results:**

Rates of outpatient consultation for circulatory system diseases were significantly lower amongst patients from Africa, Asia and Latin America compared with ethnic Norwegian psychiatric patients. Only patients from Eastern Europeans had a higher rate. With regard to hospital admission, all psychiatric patients had a lower rate than ethnic Norwegians with the exception of those from Africa where the finding was non-significant. In terms of infectious diseases, patients from African countries had significantly higher outpatient and admission rates than ethnic Norwegians. Outpatient consultation rates were lower amongst those from Western and Latin America and hospital admission rates were lower amongst those from Eastern Europe and Asia.

**Conclusions:**

The findings suggest that the majority of immigrant psychiatric patients have lower hospitalization rates for circulatory system diseases than Norwegian psychiatric patients. This may suggest that poor access for immigrants is a contributing factor, though the findings were less pronounced for infectious diseases.

## Background

An alarmingly high proportion of mental disorder patients have a higher risk of morbidity and mortality, particularly patients with severe mental disorders such as schizophrenia, bipolar disorder, or depression [1–6]. The high burden of somatic diseases is the main contributor for excess morbidity and mortality, i.e., about 60% of excess mortality is due to somatic problems [7, 8]. In particular, cardiovascular diseases (CVD) and infectious diseases (e.g., pneumonia, tuberculosis and chronic viral infections) are identified as major comorbid somatic diseases in patients with mental disorders [9–11]. For example, a review of studies reported that people with depression have a 50% greater risk of CVD [12]. The prevalence of hepatitis among people with severe mental illness is approximately 5 to 11 times higher than the estimated population rates of this infection [12, 13]. Studies have suggested that such somatic comorbidities may even be greater among immigrant psychiatric patients, particularly among refugees with post-traumatic stress syndrome and depression [14, 15].

Despite such a high burden of somatic comorbidities, rates of undiagnosed and untreated somatic illness are greater in mental disorder patients than in the general population [16]. Mental disorder patients are also subject to unacceptably high levels of disparity in healthcare access and utilization, which contribute to poor somatic health outcomes [16]. In particular, such disparities could be much worse among immigrant patients where underutilization of healthcare services is highly prevalent compared with non-immigrant patients [17, 18]. For instance, our recent research work found that the majority of immigrant groups in Norway have generally lower utilization rates of specialist mental healthcare compared to ethnic Norwegians [19]. However, there is little research investigating utilization of somatic medical services among psychiatric immigrant patients. This study therefore aims to examine the use of specialist somatic healthcare services among psychiatric patients with and without immigrant backgrounds.

Mental ill individuals in general may face a number of barriers to use and access to healthcare services, which are associated with patient- and illness-related factors as well as factors related to healthcare providers and the healthcare system. Such factors may influence the recognition and management of somatic illness. Particularly, patients with immigrant backgrounds may experience a number of such barriers. For instance, they might lack information regarding the healthcare system, little knowledge about health behaviours, or inconsistencies between their expectations and what healthcare providers are able to offer [20]. Moreover, access to care might not be straightforward, as a result of waiting lists or a tendency for lower medical referral rates amongst immigrants. Such factors can impact upon an already under-utilised system leading to significant unmet needs amongst psychiatric immigrant patients.

In this study, we therefore aimed to examine differences in the utilization of specialist somatic healthcare between immigrant and ethnic Norwegian psychiatric patients. Specifically, the study describes differences in rates of outpatient visits and hospital admission for circulatory system diseases and infectious diseases since psychiatric patients seem to be particularly affected by these disease categories. The rationale of the study is to identify utilization patterns of somatic healthcare which could suggest specific needs among immigrant psychiatric patients compared to ethnic Norwegian psychiatric patients. The empirical knowledge provided by this study will ultimately inform about inequalities in use and access to somatic healthcare, which will be vital in efforts to improve the coordination of care across the somatic and mental healthcare delivery system for vulnerable groups.

## Methods

### Study design and population

The base data file, comprising all individuals listed in the Norwegian population register per 1 January 2008 (approximately 4.7 millions), was constructed by linking socio-demographic information from Statistics Norway with data from the Norwegian Patient Registry. The analysed sample was restricted to those who had at least one contact, either outpatient or hospital admission or both, with specialist mental healthcare during 2008–2011 and were living in Norway at the start of 2008 (*N* = 329,363).

Immigrants were defined in this study as “1st generation”, i.e., born abroad by non-Norwegian parents. Because of data protection requirements, specific country background information was only available for the larger immigrant groups, while other immigrants had been pooled into broader background categories (e.g., Latin America, West Europe) by the data provider. This background information was used to construct a variable indicating five world region origins among the immigrants, in addition to Norwegians used as the reference category. Western migrants consist of those from Nordic countries, West and Central Europe, and overseas Western countries. Those from Bosnia-Herzegovina, Poland, Serbia, Russia, and other countries in these parts of Europe were classified as East European migrants. Somalia and ‘other Africa’ were combined into one category. Turkish immigrants and all migrants with an Asian background were pooled into the Asian migrant category, while the Latin America category was kept unchanged.

### Variables

The outcome variables were dichotomized and indicated whether the psychiatric patients in the study sample had attended one or more outpatient appointments or had been admitted to a somatic hospital during the four years 2008–2011, either due to infectious diseases or to diseases of the circulatory system, i.e., Chapters I and IX in the International Classification of Diseases (ICD), 10th Revision [21].

Gender was coded 0 for males and 1 for females. Information about age was only available in ten-year bands, due to data protection stipulations; the age variable (per 1 January 2008) was categorized into 0–19 years, 20–59 years, and 60 years and above.

### Statistical analysis

After describing the study population, logistic regression models were used to analyse use of specialist somatic healthcare during 2008–2011 among those with a Norwegian background and the immigrants classified according to world regions of origin. Estimates were adjusted for age and gender. The results from these regression models are reported as marginal effects (predicted probabilities with robust standard errors – β (se)). Reporting marginal effects makes interpretation more easy since the marginal effects indicate average change in the probability of the outcome (P (y = 1)) when taking into account the distribution of the other independent variables across all observations. The reported results denote the predicted probability of having had at least one contact with specialist somatic healthcare (either outpatient, or hospital admission) because of conditions of the circulatory system or because of infectious diseases during the study years. Estimates were judged as statistically significant when *p*-values ≤0.05. The analyses were made by means of Stata SE/14.

## Results

### Characteristics of the study population

Table 1 illustrates that, of the total number of psychiatric patients (*N* = 329,363) there was a higher proportion of children and adolescents aged 0–19 and a lower proportion of those aged 60 and above in the immigrant sample, compared to the sample of ethnic Norwegians. About half of immigrant psychiatric patients were from Western countries. In total, about 8% of the ethnic Norwegians and 4% of the immigrants had outpatient contact with specialist somatic healthcare for circulatory system diseases at least once during the years 2008–2011. The rates of outpatient contacts for infectious diseases were comparable between Norwegians and immigrants. In both types of disease categories, immigrant psychiatric patients had lower hospital admission rates than ethnic Norwegian psychiatric patients.

**Table 1:** Descriptive summary of the study population – patients who had contacts with psychiatric specialist healthcare from 2008 to 2011, *N* = 329,363

Variables	Ethnic Norwegians (N = 276,890, 83.3%)		Immigrants (N = 52,473, 12.7%)	
	N	%	N	%
Age				
0–19	91,001	32.87	27,326	52.08
20–59	154,707	55.87	24,587	46.86
60+	31,182	11.26	560	1.07
Gender				
Male	129,275	46.69	26,559	50.61
Female	147,615	53.31	25,914	49.39
World regions of origin				
Norway	276,890	100	–	–
Western countries	–	–	29,156	55.56
East Europe	–	–	5507	10.49
Africa	–	–	3035	5.78
Asia	–	–	12,065	22.99
Latin America	–	–	2710	5.16
Circulatory system diseases				
Outpatient visit rate	21,559	7.79	2241	4.27
Hospital admission rate	14,284	5.16	917	1.75
Infectious diseases				
Outpatient rate	7228	2.61	1461	2.78
Admission rate	7411	2.68	997	1.90

### Utilization of specialist somatic healthcare services across world regions of origin

Table 2 presents utilization rates among Norwegians and immigrants from the five world regions of origin, i.e., the proportions (in %) who made contact at least once during 2008–2011. It indicates that Norwegians and Eastern European psychiatric patients had a higher proportion of outpatient visits for circulatory system diseases, while immigrants with African origins had a higher proportion of hospital admission for circulatory system diseases. For infectious diseases, there were less marked differences as to the outpatient visit, while Norwegians had a higher rate of hospital admissions.

**Table 2:** Proportion of the use of specialist healthcare for diseases of circulatory and infectious diseases among psychiatric patients from 2008 to 2011

Region origin	Circulatory system diseases			Infectious diseases				
	Outpatient		Admission	Outpatient		Admission		
	N	%	N	%	N	%	N	%
Norway	21,559	7.79	7228	2.61	7411	2.68	14,284	5.16
Western countries	1346	4.57	800	2.71	633	2.15	596	2.02
East Europe	347	6.03	154	2.68	89	1.55	130	2.26
Africa	97	3.11	141	4.52	80	2.56	50	1.60
Asia	701	4.92	390	2.74	229	1.61	300	2.11
Latin America	55	2.03	85	3.14	52	1.92	19	0.70

Marginal effects (predicted probabilities) for each world region of origin, age- and gender-adjusted, estimated by logistic regression models, are presented in Tables 3-4. These predicted values indicate the probability of outpatient visit and admission to specialist somatic hospitals among specific world regions of origin.

**Table 3:** Logistic regression estimates (marginal effects) showing age- and gender-adjusted predicted probabilities for the use of specialist healthcare for circulatory system diseases during 2008–2011 among psychiatric patients

Regions of origin	Specialist healthcare for circulatory system diseases			
	Outpatient		Admission	
	β (se)	95% CI	β (se)	95% CI
Norway	0.073 (0.001)	0.072–0.074	0.047 (0.001)	0.046–0.048
Western countries	0.064 (0.001)	0.061–0.067	0.036 (0.001)***	0.033–0.038
East Europe	0.080 (0.004)***	0.072–0.087	0.038 (0.003)**	0.032–0.044
Africa	0.053 (0.005)***	0.043–0.063	0.037 (0.005)	0.028–0.046
Asia	0.066 (0.002)**	0.061–0.071	0.036 (0.002)***	0.032–0.039
Latin America	0.040 (0.005)***	0.030–0.050	0.021 (0.004)***	0.012–0.029

Statistically significance values showing differences between immigrants and the Norwegians (the reference group): *p < 0.05, **p < 0.01, ***p < 0.001. β (se) = predicted probabilities and robust standard error (in parenthesis) and are adjusted to age and gender. *CI* = confidence interval

**Table 4:** Logistic regression estimates (marginal effects) showing age- and gender-adjusted predicted probabilities for the use of specialist healthcare for infectious diseases during 2008–2011 among psychiatric patients

Regions of origin	Specialist healthcare for infectious diseases			
	Outpatient		Admission	
	β (se)	95% CI	β (se)	95% CI
Norway	0.026 (0.001)	0.025–0.027	0.026 (0.001)	0.025–0.027
Western countries	0.028 (0.001)*	0.026–0.030	0.026 (0.002)	0.023–0.028
East Europe	0.027 (0.002)	0.023–0.032	0.018 (0.001)**	0.014–0.021
Africa	0.047 (0.004)***	0.039–0.055	0.034 (0.004)*	0.027–0.041
Asia	0.028 (0.001)	0.025–0.031	0.019 (0.001)***	0.016–0.021
Latin America	0.033 (0.003)*	0.026–0.041	0.027 (0.003)	0.020–0.035

Statistically significance values showing differences between immigrants and Norwegians (the reference group): *p < 0.05, **p < 0.01, ***p < 0.001. β (se) = predicted probabilities and robust standard error (in parenthesis) and are adjusted to age and gender. *CI* = confidence interval

In Table 3, for circulatory system diseases, East European psychiatric patients had significantly higher outpatient visits than the ethnic Norwegian psychiatric patients, while those with Africa, Asia and Latin America backgrounds had a significantly lower outpatient visits. As to the rate of admission for circulatory system diseases, all psychiatric immigrant patients had significantly lower rates compared to Norwegian psychiatric patients, except for those from Africa who did not differ significantly from the Norwegian psychiatric patients.

Predicted probabilities in Table 4 indicate that African psychiatric patients had significantly higher outpatient visits and admission rates for infectious diseases than the Norwegian psychiatric patients. Psychiatric patients from western countries and Latin America had also a higher outpatient visit for infectious diseases as compared to the Norwegian psychiatric patients. However, psychiatric patients with East Europe and Asia backgrounds had lower rates of hospital admission for infectious diseases than the Norwegian psychiatric patients.

## Discussion

Our study found that the majority of non-Western immigrant patients have lower rates of utilization of somatic healthcare services for diseases of the circulatory system. In particular, the rate of hospitalization was significantly lower in most immigrant groups, except for psychiatric patients with African backgrounds. Prior research findings have revealed that immigrant psychiatric patients, particularly refuges and asylum seekers, have a higher burden of somatic comorbidities, e.g., hypertension, diabetes and cardiovascular diseases [14, 15, 22, 23]. Based on this evidence, we suggest that underutilization is likely among non-Western immigrants, in the sense that comorbidities between mental disorders and circulatory system diseases that would normally trigger specialist healthcare for Norwegians, do not lead to specialist healthcare for many non-Western immigrants. Specifically, it seems that under-utilisation of somatic healthcare services is unlikely to be due to lack of availability or need. This may lead to lack of detection or treatment of circulatory system diseases in immigrant psychiatric patients, or late diagnosis resulting in poor prognosis, treatment responses and outcomes [9, 24, 25].

In contrast, we found less disparities in the utilization rates of somatic specialist healthcare for infectious diseases, especially for the outpatient services. The findings rather reveal that African psychiatric patients had higher outpatient and hospital admission rates for infectious diseases. This could be due to the heightened attention given to screening on arrival and management of infectious diseases among immigrants, especially for those from high burden countries, e.g., African immigrants. Such increased attention from both health personnel and policy makers towards prevention and management of infectious diseases could contribute in reducing inequalities in access and utilization of somatic care for infectious diseases as well as improve coordination of care across the somatic and mental healthcare delivery system.

This study indicates that immigrants from non-Western countries are especially faced with a double barrier to health care. As shown in an earlier paper, immigrants have less utilization of mental health services in general [19], and those who access mental health services have a higher barrier to get somatic services. There is an urgent need to inform policy makers and health care workers about these disparities and address them accordingly.

One strength of the present study is that it has investigated use of specialist somatic healthcare among immigrants with high-quality register data which cover the entire population, implying that selection bias is practically absent. Only a very small number of private-practising physicians, psychologists, psychiatrists and hospitals are not obliged to report on their activities to the Norwegian Patient Registry. Thus, the patients analysed here will cover practically all patients in specialist psychiatric care during the study years, and the estimated utilization rates in specialist somatic healthcare are likely to be quite precise. Another strength is that the data provided by Statistics Norway include all registered immigrants in Norway, meaning that each immigrant world origin was represented by a sample of usable size. Further, the registry offers data concerning the origin of patients making it possible to explore based on world regions of origin rather than heterogeneous groups, i.e., western vs. non-western immigrants or immigrants from high vs. low income countries. The study period 2008–2011 coincides with the ‘Great Recession’ in Europe which was followed by austerity measures in the health care of many countries. However, since the international financial crisis had few effects in Norway, the time period analysed here did hardly differ from previous or later years.

Various limitations may nevertheless have affected the reported results of this study. The outcome variables indicate whether the psychiatric patients had at least one outpatient visit or at least one hospital admission for circulatory conditions or infectious diseases during the observation period, but they do not distinguish between few and many visits and hospital stays. Only registered inhabitants are included on the register, thereby, migrants staying in Norway without being registered, and asylum seekers whose applications have not been decided on, are not part of the analyzed samples, implying that particularly vulnerable migrants are not analysed here. A weakness which affects the present study as well as most other investigations based on administrative registers, is that the information available is often very limited and possibilities for testing hypotheses and detailed explanations will be restricted. Research of the themes addressed in this study will benefit from data more suitable for exploring explanatory mechanisms.

## Conclusions

This study has served to highlight significant differences in the uptake of somatic healthcare services for circulatory diseases between psychiatric patients based upon their world regions, though there are less variations for infectious diseases. Underutilisation of hospitalisation is most likely amongst patients from Asia and Latin America. The findings suggest that interventions addressing inequalities in access to and utilization of somatic healthcare should primarily target non-Western psychiatric immigrant patients with diseases of the circulatory system. Policy-makers and service providers should try to implement measures which improve the responsiveness of somatic healthcare services to the needs of psychiatric immigrant patients. Such measures should emphasize a much stronger follow up of somatic disorders in mental health care and an improved cooperation between mental and somatic health care.

## Abbreviation

CVD: Cardiovascular diseases
ICD: International classification of diseases
